# Current trends and spatial-temporal dynamics of veterinary dentistry research: A scientometric study

**DOI:** 10.14202/vetworld.2024.666-671

**Published:** 2024-03-21

**Authors:** Daniel Alvitez-Temoche, Elca del Aguila, Diego Galarza-Valencia, Iván Calderón, Fran Espinoza-Carhuancho, Josmel Pacheco-Mendoza, Frank Mayta-Tovalino

**Affiliations:** 1 Unidad de Investigación, Innovación y Emprendimiento, Faculty of Dentistry, Universidad Nacional Federico Villarreal, Lima 00051, Peru; 2 Department of Academic, Faculty of Dentistry, Universidad Nacional Mayor de San Marcos, Lima 00051, Peru; 3 Grupo de Bibliometría, Evaluación de evidencia y Revisiones Sistemáticas (BEERS), Human Medicine Career, Universidad Cientifica del Sur, Lima 00051, Peru; 4 Vicerrectorado de Investigación, Universidad San Ignacio de Loyola, Lima 00051, Peru

**Keywords:** bibliometrix, scientometrics, veterinary dentistry

## Abstract

**Background and Aim:**

Understanding dental care in dogs has made remarkable progress in veterinary medicine. Therefore, this study aimed to analyze the academic literature published in veterinary dentistry from 1990 to 2023.

**Materials and Methods:**

A descriptive study was conducted using a scientometric approach and metadata from the Web of Science database. A search strategy adapted for this database was developed using MeSH and Emtree terms and the Boolean operators AND and OR. Using Bibliometrix, different metrics were evaluated to assess the scientific production of researchers and institutions and the impact of authors based on their publications. CiteSpace was also used for co-citation analysis and visualization of citation networks, trends, and patterns in this field of study over time.

**Results:**

The bibliometric study analyzed 211 documents from 50 different sources from 1990 to 2023, with an annual growth rate of 6.5%, covering the period 1990–2023. A total of 474 authors were identified, with an average of 2.82 coauthors per paper and 11.85% international coauthorships. The average age of the papers was 12.4 years and 4.55 citations per paper. The most common types of documents were articles (154 documents).

**Conclusion:**

Research in veterinary dentistry has shown steady growth from 1990 to 2023. Although there have been fluctuations in article production over the years, there has been a steady growth in article production in veterinary dentistry in general. The annual average number of citations per article has varied over the years, reaching 45 in 2015. However, the average number of citations per article has decreased significantly from 2021 to 2023.

## Introduction

The importance of maintaining good dental hygiene in animals is comparable to that in humans. Daily tooth brushing is the gold standard for prophylaxis and prevention of periodontal disease [1, 2]. These preventive measures are not only necessary for oral health but also important for the animal’s overall well-being. Periodontal diseases may have systemic consequences that affect other organs and body systems. Therefore, to protect the health and improve the quality of life of canines, it is essential to integrate daily brushing into their routine [3]. In veterinary medicine, remarkable progress has been made in understanding dental care for dogs. Research on groups of dogs has provided essential information on the optimal frequency of tooth brushing. Increasing the frequency of daily brushing helps slow periodontal disease progression [4]. This highlights the need for a veterinarian to perform a personalized assessment of the dental health of each dog and adapt the oral hygiene plan to the needs of each dog. Prevention and early intervention are necessary to prevent more serious problems and ensure the complete well-being of pets [5].

In pet dentistry, it is essential to prevent oral conditions in dogs to ensure their quality of life. Daily tooth brushing is a key preventive strategy because it is impossible to predict which dogs may suffer from periodontal diseases. Whereas gingivitis, which manifests with gum inflammation, is reversible through proper oral care, periodontitis is an irreversible condition involving tooth support loss and can lead to tooth loss. Therefore, it is vital that dog owners, in collaboration with their veterinarians, prioritize the prevention of these pathologies because the management of advanced stages is more complex and has a less encouraging prognosis than prevention [6]. In animal dentistry, there is a particular challenge: to ensure that pet owners correctly follow tooth-brushing guidelines for dogs. It is common for veterinarians to overestimate adherence to recommended oral hygiene plans, a situation similar to medicine for humans, where adherence to medical treatment does not exceed 50%. In contrast, 85% of Swedish citizens brush their teeth twice daily, indicating that the quality of information and the effectiveness of communication from healthcare personnel are crucial to the success of home dental care [7, 8].

Although little research has been conducted on how dog owners process and implement dental recommendations, how veterinarians and veterinary technicians communicate can significantly impact owners’ engagement with their pets’ dental cleanliness. Validated surveys have been proven effective for assessing behaviors and opinions related to this topic. Therefore, to ensure the oral health of canines [9–11], veterinarians must emphasize improving their communication and educational skills regarding preventive dental care. However, progress in the creation of simulations is remarkable. With this advancement, it becomes imperative to investigate how they influence the learning and skill acquisition of dental and veterinary students to understand the impact on their training [12]. Implementing simulations using virtual reality (VR) and haptic technology has transformed teaching in human dentistry, placing it ahead of veterinary medicine in terms of technological innovations [13]. It should be emphasized that VR simulators provide objective assessment and instant feedback, which could be more effective than human expert assessment [14]. However, instructor feedback is indispensable to point out areas of improvement in students’ technique and provide context to errors made during dental procedures, thus emphasizing the need for further studies [15, 16].

Therefore, this study aimed to perform a scientometric analysis of the current trends and spatiotemporal dynamics of research in veterinary dentistry and evaluate its impact on scientific research in this field.

## Materials and Methods

### Ethical approval

Because this is a scientific study using open access data, no ethical implications are presented.

### Study period and location

Data were extracted on 7/12/23. Analyses were performed at the Vicerrectorado de Investigacion, Universidad San Ignacio de Loyola, Lima, Peru.

### Study design

A descriptive study with observations was conducted using a scientometric approach. Different quantitative methods were used to analyze the academic literature published on the subject. Metadata from the Web of Science database (Clarivate Analytics) were used from 1999 to 2023.

### Search strategy

First, the specific subject of the search was detailed, and a search strategy adapted for the Web of Science was developed. MeSH and Emtree terms were used. The Boolean operators AND and OR were also used. The download was performed on December 07, 2023, and 211 metadata were found with the following search strategy: TS = (“veterinary dentistry” OR “oral veterinary medicine” OR “veterinary oral surgery” OR “endodontics” OR “animal oral health” OR “animal dental health” OR “animal dental diseases” OR “animal dental treatment” OR “animal dental surgery” OR “dog dentistry” OR “cat dentistry”) AND SU = (Veterinary). Excluded are editorials (25), reviews (17), early access (7), and letters (6).

### Bibliometric indicators

Different metrics were evaluated to assess the scientific production of investigators and institutions, the impact of the authors from their publication using Bibliometrix (R version 4.3.2, https://www.r-project.org/). On the other hand, CiteSpace (6.3.R.1, https://citespace.podia.com/) was also used for the analysis of the co-citation and visualization of the networks of cites and tendencies and patterns in this field of study throughout the study period.

### Statistical analysis

Different metrics were evaluated to assess the scientific production of investigators and institutions and the impact of the authors on their publication using bibliometrix. CiteSpace was also used to analyze the co-citation and visualization of the redes of cites and tendencies and patterns in this field of study throughout the study period.

## Results

The bibliometric study covered the period 1990–2023 and analyzed 211 documents from 50 different sources with an annual growth rate of 6.5%. A total of 474 authors were identified, with an average of 2.82 coauthors per paper and 11.85% international coauthorships. The average age of the papers was 12.4 years and 4.55 citations per paper. The most common types of documents were articles (154 documents) (Table-1).

**Table-1:** Scholarly output.

Description	Results
Timespan	1990:2023
Sources (Journals, Books, etc.)	50
Documents	211
Annual growth rate %	6.5
Document average age	12.4
Average citations per doc	4.55
References	4754
Document contents	
Keywords plus (ID)	423
Author’s keywords (DE)	469
Authors	
Authors	474
Authors of single-authored docs	63
Authors collaboration	
Single-authored docs	86
Co-authors per Doc	2.82
International coauthorships %	11.85
Document types	
Article	154
Article; early access	6
Correction	1
Editorial material	25
Letter	6
News item	1
Note	1
Review	16
Review; early access	1

**Figure-1:**
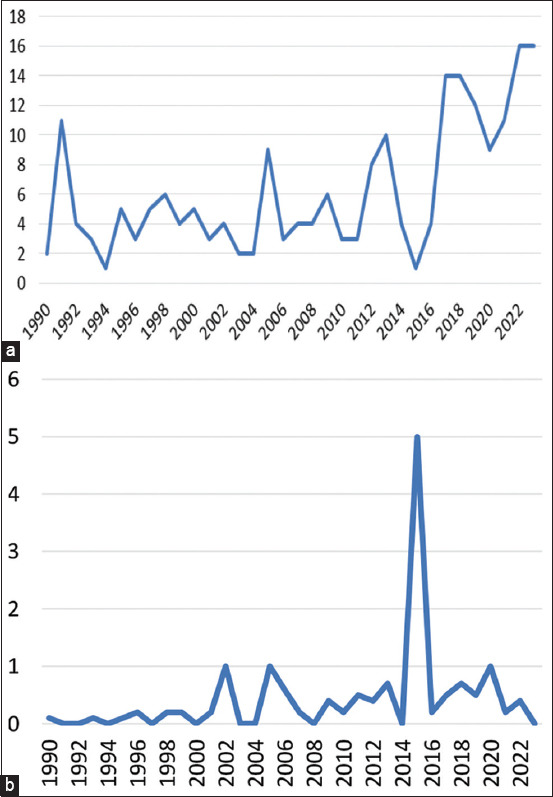
(a and b) Annual scientific production and average citations per year.

From 1990 to 2023, annual article production increased. It started with only 2 articles in 1990 and increased to 16 by 2023. A significant increase in article production started in 2017, with 14 articles produced that year, which continued until 2023. Although there were fluctuations in article production over the years, there was a steady growth in article production in veterinary dentistry in general (Figure-1a). The average annual number of citations per article varied over time. In 1990, the average number of citations per article was 4. A notable peak occurred in 2002 and 2005, with an average of 22.5 and 20 citations per article, respectively. The year with the highest average number of citations per article was 2015, with 45 citations per article. However, the average number of citations per article decreased significantly from 2021 to 2023, reaching 0 in 2023 (Figure-1b).

**Figure-2:**
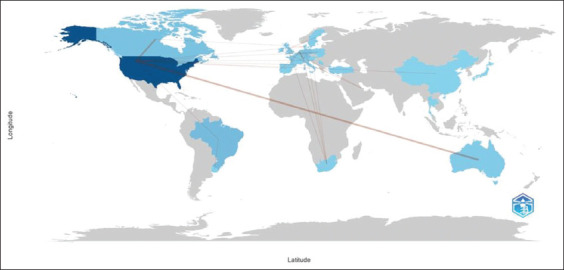
Country collaboration map.

The cooperation map between countries showed several interactions between different countries. For example, the United States cooperated with Australia on four occasions and Canada on three occasions. Germany cooperated with Belgium, South Africa, and Switzerland once. The Netherlands cooperated with France and Portugal once and with Switzerland twice. International collaboration is a common practice in veterinary dentistry, indicating a globalized field of study (Figure-2).

**Figure-3:**
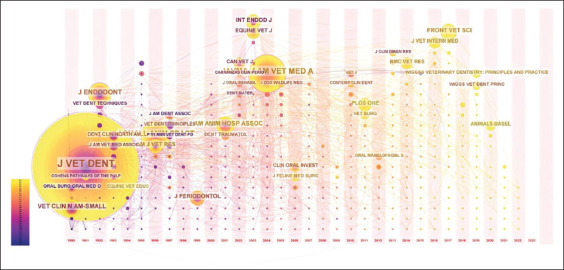
Time-zone visualization.

The “Time-zone visualization” graph allowed for visualizing scientific journal citations using a timeline. Since 1991, J Vet Dent has been the most cited journal. In contrast, a great interaction in citations was observed, especially in 1996, 1997, and 2004; however, citations decreased in the last few years after 2018 (Figure-3).

**Figure-4:**
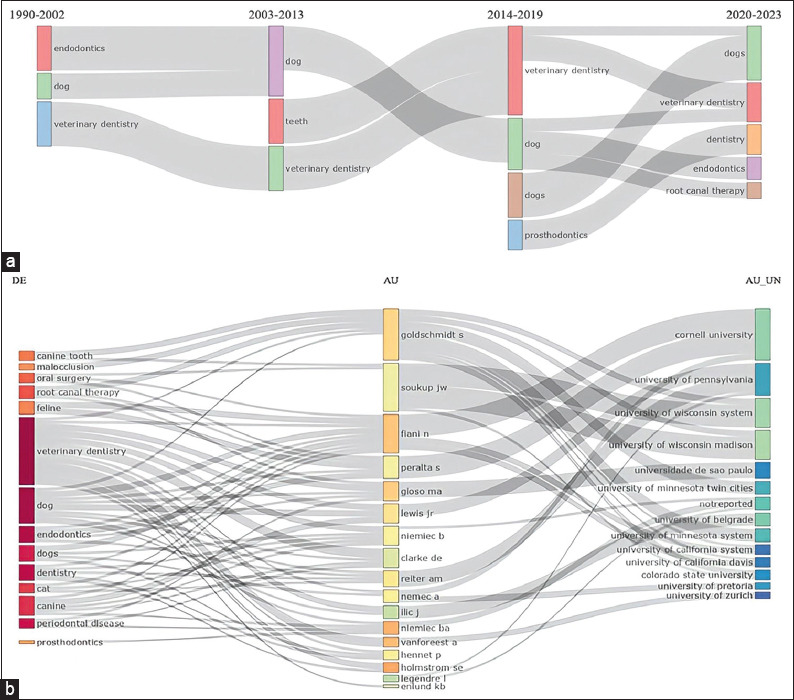
(a and b) Thematic evolution and three-field plot.

When evaluating the thematic evolution of the most important thesauri, it became evident that the word “veterinary dentistry” was the most predominant from 1990 to 2022, even up to 2020–2023. Second, “endodontics” (1990–2002) was strongly interrelated with “dog” (2003–2013), “dog” (2014–2019), “veterinary dentistry,” “endodontics,” and “root canal therapy” (2020–2023) (Figure-4a). In contrast, the three-field plot evidenced an important relationship between the thesaurus “veterinary dentistry,” which was the most used keyword by most authors such as Peralta S, Niemiec B, and Clarke DE, and these, in turn, were related to Cornell University, the University of Pennsylvania, and the University of Wisconsin Madison, among others (Figure-4b).

**Figure-5:**
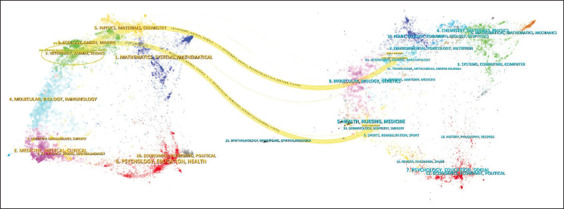
Dual-map overlay.

The “Dual-map overlay” graph allowed for analyzing the structure and trends in this field of study, representing the disciplines on the left and the citation destination on the right. Thus, patterns of connection were found between Cluster 5 (physics, materials, chemistry) and Cluster 7 (veterinary, animal, science) that ended up being cited in Cluster 11 (veterinary, animal, parasitology) and Cluster 14 (dermatology, dentistry, surgery), which could indicate a strong interaction or influence between these disciplines (Figure-5).

**Figure-6:**
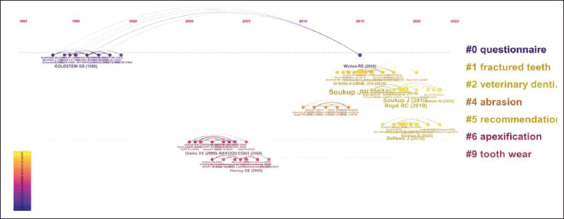
Timeline view cluster.

When analyzing the structure and trends in this field of study, it was possible to identify the clusters of the most cited documents over time. In 1990, Goldstein corresponded to Cluster 0 (questionnaire). In contrast, in Cluster 1 (fractured teeth), Walton RE 2015 was the most representative, indicating that the topic related to that cluster is gaining popularity (Figure-6).

## Discussion

It is fascinating to know how specific and essential brushing techniques applied in animals can effectively delay plaque and tartar accumulation. Frequent brushing, intended to clean the tooth surface mechanically, can reduce plaque and tartar accumulation. These findings suggest that brushing has a greater impact on tartar than plaque. Although veterinary dentistry is a relatively new field, all techniques have been applied, and animals are often used to test materials and procedures before their implementation in human clinical practice. Note that veterinary dentistry is not limited to dogs and cats. Small animals, such as rabbits and rodents, may also require dental care [17].

Dental care for dogs is very important in the field of laboratories. In daily brushing, a specific brushing protocol implemented by trained technicians using a particular type of toothbrush results in a statistically significant reduction in the average plaque and tartar score. However, another study found that less frequent brushing was less effective, and there was no significant difference in the effectiveness of plaque removal compared to other medical controls. Veterinary dentistry services may include several procedures, including oral examination, dental cleaning, tooth extraction, grafting, and whitening. In addition, veterinary dentists can also take care of more serious problems, such as dental and maxillofacial fractures and oral tumors [18].

Periodontal disease, a common chronic infection in humans and dogs, may contribute to other conditions, such as heart disease and stroke. Although a direct connection between periodontal disease and these other conditions has not yet been demonstrated in most cases, several theories could explain this association [19]. Periodontitis is generally a persistent or recurrent condition that may last for a lifetime. In these cases, frequent episodes of bacteremia or the presence of bacteria in the blood are likely due to small lesions in the periodontal area. These episodes may lead to infections in other body parts [20].

Despite the progress made, veterinary dentistry is faced with significant challenges. Low awareness among pet owners about the relevance of oral health in animals and the limited access to specialized services in certain areas highlights the need to address not only technical aspects but also socio-educational factors [21].

This study explored recent advances in veterinary dentistry, emphasizing the essential role of dental prevention and treatment [22, 23] in animal welfare. By analyzing current challenges, from the lack of awareness to restrictions in access to specialized services, this study sought solutions and strategies to improve dental care for animals, thus contributing to a healthier and happier life for our pets. The following limitations were identified in our study. First, the selection of databases may impact the research results. Relevant studies published on other platforms may be overlooked. Second, a linguistic bias could arise if the study is restricted to publications in certain languages (such as English). Third, the selection of search terms may affect the results. Some relevant studies could be missed if the search terms were too specific. Finally, research trends may vary over time, which could affect the relevance and applicability of our findings.

## Conclusion

This study revealed that research in veterinary dentistry showed sustained growth from 1990 to 2023, with an annual increase of 6.5%. In addition, international collaboration is a common feature in this field, suggesting a globalized study approach. Regarding thematic evolution, the term “veterinary dentistry” has become the most prominent term over time. Patterns of connection among various disciplines have been identified through the dual-map overlay analysis, which could indicate a significant interaction or influence between these disciplines. This suggests that veterinary dentistry is an interdisciplinary field that benefits from advances in other areas. Although the average number of citations per article has decreased in recent years, it does not detract from the importance of research in this field.

## Authors’ Contributions

DAT, FMT, FEC, IC, DGV, EA, and JPM: Conception of the study. FMT, JPM, DAT, and FEC: Extracted, verified, and analyzed the data and drafted and revised the manuscript. All authors have read, reviewed, criticized, and approved the final manuscript.
